# Citrus Extract Improves the Absorption and Utilization of Nitrogen and Gut Health of Piglets

**DOI:** 10.3390/ani10010112

**Published:** 2020-01-10

**Authors:** Yiyan Cui, Zhimei Tian, Gang Wang, Xianyong Ma, Weidong Chen

**Affiliations:** 1 Institute of Animal Science, Guangdong Academy of Agricultural Sciences, Guangzhou 510640, China; cuiyiyan@gdaas.cn (Y.C.); tianzhimei@gdaas.cn (Z.T.); wanggang@gdaas.cn (G.W.); 2 State Key Laboratory of Livestock and Poultry Breeding, Guangzhou 510640, China; 3 Key Laboratory of Animal Nutrition and Feed Science in South China, Ministry of Agriculture, Guangzhou 510640, China; 4 Guangdong Key Laboratory of Animal Breeding and Nutrition, Guangzhou 510640, China; 5 Guangdong Engineering Technology Research Center of Animal Meat Quality and Safety Control and Evaluation, Guangzhou 510640, China

**Keywords:** citrus extract, piglet, plasma free amino acids, intestinal morphology, intestinal enzymes, ammonia nitrogen, nitrogen, phosphorus

## Abstract

**Simple Summary:**

Weaning can cause weaning stress and reduce the growth performance of piglets. Citrus extract has strong anti-oxidant and anti-inflammatory effects which can improve animal health. The aim of this study was to evaluate the efficacy of citrus extract as a substitute for antibiotics in piglet diets. The results of this study indicate that citrus extract increased the concentrations of plasma essential amino acids, improved intestinal morphology and digestive enzymes activity.

**Abstract:**

The purpose of this study was to investigate the effects of citrus extract (CE) on plasma free amino acids, intestinal morphology and enzymes activity, fecal nitrogen and phosphorus emissions in piglets. The experiment was performed on 144 weaned piglets (Duroc × Landrace × Large White) divided into three groups. Control (CON), fed a basic diet; Antibiotic (ANTI), fed a basic diet supplemented with 75 g/t chlortetracycline; Citrus extract (CE), fed a basic diet supplemented with 300 mL/t CE. The albumin content of the CE group was significantly higher than the CON group. Compared with the CON and ANTI groups, the CE group had increased concentrations of plasma total essential amino acids and threonine. Compared with the CON group, CE increased the α-aminoadipic acid concentration, while compared with ANTI group, it increased the 3-methylhistidine concentration. Compared with the CON group, the crypt depth of duodenum, jejunum and ileum decreased, and the ratio of villus height to crypt depth of ileum increased in the ANTI and CE groups. CE increased the activity of alkaline phosphatase and lipase in duodenum, and the activity of alkaline phosphatase and trypsin in jejunum. In brief, CE improved the absorption and utilization of nitrogen, intestinal morphology and digestive enzymes activity.

## 1. Introduction

Weaning changes the physiology of animals and can lead to intestinal dysfunction [1]. While diarrhea is not the only symptom of intestinal dysfunction after weaning, digestive functions are the most impacted by weaning. Antibiotics are commonly used to treat these conditions, to prevent diarrhea, promote growth, and improve intestinal digestion and absorption. However, the overuse of antibiotics can result in drug-resistant bacteria and environmental pollution. In addition, animal manure emits a large amount of noxious gas, nitrogen (N), and phosphorus (P), which can have adverse effects on animal and human health and may be increased by intestinal distress. Finding an alternative to antibiotics that reduces both antibiotic use and N emissions, improves animal growth, benefits farms economically, and reduces environmental pollution would benefit multiple sectors of society.

In general, nutrient excretion can be reduced by avoiding excessive feeding of specific nutrients or enhancing nutrient use by the animal through nutrient operations [2]. Citrus flavonoids are reported to be the most biologically active compounds on Earth [3]. Hesperidin, for example, can prevent intestinal inflammation in mice [4]. Citrus extract (CE) has various biological functions, including anti-cancer [5], anti-bacterial [6,7], anti-oxidant [7,8], and anti-inflammatory [9,10]. Its strong anti-oxidant and anti-inflammatory effects are especially valuable in improving animal health [9,11]. Animal experiments have shown that orange peel extract improved immune response and disease resistance of broilers without affecting their average daily gain (ADG), average daily feed intake (ADFI), and feed conversion rate [12]. Citrus flavonoids may prolong the shelf life of eggs, appear to possess anti-inflammatory properties and could improve the yolk color without having any side effects on the performance or egg quality traits [9]. Citrus purified bioactive compounds have also been shown to improve the anti-oxidant capacity of plasma and meat in sheep [13]. Our own studies have shown that CE improved the anti-oxidant capacity and immune function of piglets [14].

Immunization stimulates a change in the utilization of amino acids (AA), which leads to reduced productivity in pigs by repurposing AA away from protein retention for use in immune responses [15]. Plasma-free amino acids (PFAA) are a primarily source of AA used for muscle protein biosynthesis, a key indicator of protein turnover in the body [16]. We hypothesized that CE benefits the intestinal ammonia nitrogen (AN) content as well as the fecal N and P emissions in piglets by improving protein (AA) metabolism and intestinal health. Therefore, this study evaluated the effects of CE supplementation on plasma biochemistry, PFAA, intestinal morphology and digestive enzymes activity, AN content, and fecal N and P emissions in piglets.

## 2. Materials and Methods

This study was carried out in accordance with the Guiding Suggestions About Treating Experimental Animals Amicably of the Science and Technology Ministry of China (2006, Document no. 398, China). Animal procedures experiments were approved by the Animal Care and Use Committee of Guangdong Academy of Agricultural Sciences (authorization number GAASIAS-2017-11-17).

### 2.1. Materials

Chlortetracycline was purchased from Guangdong Newland Feed Science & Technology Co., Ltd. CE was provided by Guangdong Runsen Environmental Technology Development Co., Ltd. The primary active ingredients of CE are the total flavonoids (20.77%), vitamin C (3.04%), citric acid (2.89%), and vitamin E (2.38%).

### 2.2. Experimental Design, Animals and Diets

A total of 144 crossbred piglets (Duroc × Landrace × Large White) weaned at 28 days of age (8.39 ± 0.10 kg) were used. Piglets were assigned to one of 3 dietary treatments. Each treatment comprised 6 pens, with 8 pigs (4 barrows and 4 gilts) in each. Control pigs (CON) were fed a corn-soybean meal basal diet, antibiotic pigs (ANTI) were fed the basal diet supplemented with 75 g/t chlortetracycline, the others were fed the basal diet supplemented with 300 mL/t citrus extract (CE). All experimental diets were formulated to meet the nutrient requirements suggested by the NRC (2012; Table 1). All piglets had ad libitum access to feed and water throughout the 28-day experimental period.

### 2.3. Sample Collection and Slaughter Procedure

During the experiment, the piglets body weight (BW) were measured at 0 d (start) and 28 d (finish) of the experiment to calculate the average daily gain (ADG). The feed consumption was measured every day to calculate the average daily feed intake (ADFI) and feed to gain ratio (F/G). Fresh fecal grab samples were collected in the morning during the last week (experimental d 21–28) and pooled by pen and stored at −20 °C for N and P emissions analysis. Each piglet was weighed at the end of the experiment, the two pigs closest to the average weight of treatments were selected from each replicate pen for slaughter. Approximately 12 h before slaughter, feed was withheld, but water remained available. Blood samples (sodium-EDTA) were allowed to rest at room temperature for a few minutes and centrifuged at 1800× *g* at 4 °C for 10 min to extract the plasma which was then frozen at −80 °C until analysis. Animals were maintained under general anesthesia and intravenously euthanized via a jugular injection of 4% sodium pentobarbital solution (40 mg/kg BW). For intestinal morphology examination, samples of the duodenum (middle), jejunum (a segment of the small intestine 2–3 m proximal to the duodenum), and ileum (a segment of the small intestine 1 m proximal to the ileocecal junction) were dissected out and immediately put into 4% paraformaldehyde. A section of duodenum and jejunum were washed with phosphate buffer saline, and immersed quickly in liquid nitrogen, before stored at −80 °C for digestive enzymes activity analysis. The digesta in the jejunum and ileum were collected and immediately immersed in liquid nitrogen and stored at −80 °C for AN analysis.

### 2.4. Plasma Biochemistry

Plasma urea nitrogen (PUN, C013-2-1, urease method), albumin (A028-1-1, colorimetry), and total protein (TP, A045-2-1, coomassie brilliant blue method) were measured using assay kits according to the manufacturer’s instructions (Nanjing Jiancheng Bioengineering Institute, Nanjing, China). The plate was read by a multi-functional enzyme labeling instrument (Spectra Max M5, Molecular Devices, San Jose, CA, USA) at 640 nm (PUN), 628 nm (albumin), and 595 nm (TP). There were 3 duplicates for each plasma sample, and 12 replicates in each group.

### 2.5. Urea, NH3, and PFAA

The amounts of urea, NH_3_, and PFAA were determined using a post-column derivatization of ninhydrin. Exactly 400 μL plasma was absorbed and 1.2 mL 10% sulfosalicylic acid was added and mixed fully. Then, it was centrifuged at 12,000× *g* for 15 min at 4 °C. The supernatants were filtered with 0.22 μm filters for testing. The urea, NH_3_, and PFAA concentrations were measured with an automatic amino acid detector equipped with sodium ion exchange column (L-8900, HITACHI, Tokyo, Japan). There were 3 duplicates for each plasma sample, and 12 replicates in each group.

### 2.6. Intestinal Morphology

The procedure for determining of intestinal morphology was as follows. The intestinal samples were dehydrated, embedded, sectioned, hematoxylin-eosin stained, observed under microscope, and scanned. The Pannoramic Viewer version 1.15.3 software (3DHISTECH, Budapest, Hungary) was used to measure the villus height (VH), crypt depth (CD), and villus height to crypt depth ratio (VCR), and 8 replicates of complete and upright villus from each histological section were selected for measurement. Villus height was defined as the distance from villus base to tip, and crypt depth as the distance from villus base to lamina muscularis mucosae. There were 12 histological sections in each group.

### 2.7. Intestinal Digestive Enzymes Activity

The activity of digestive enzymes was measured after homogenization of duodenum and jejunum tissue in 0.9% saline at 1:9 (m:v), at 1500 r/min for 2 min, followed by centrifugation at 1800× *g* and 4 °C for 15 min. The supernatant was collected and then the activities of α-amylase (C016-1-1, starch-iodine colorimetry), lipase (A054-1-1, colorimetry), sucrose (A082-2-1, colorimetry), maltase (A082-3-1, colorimetry), pepsin (A080-1-1, colorimetry), trypsin (A080-2-2, ultraviolet colorimetry), and alkaline phosphatase (AKP, A059-1-1, colorimetry) were determined according to kit instructions (Nanjing Jiancheng Bioengineering Institute, Nanjing, China). The plate was read by a multi-functional enzyme labeling instrument (Spectra Max M5, Molecular Devices, San Jose, CA, USA) at 660 nm (α-amylase and pepsin), 420 nm (lipase), 505 nm (sucrose and maltase), 253 nm (trypsin) and 520 nm (AKP). There were 3 duplicates for each homogenate sample, and 12 replicates in each group.

### 2.8. Ammonia Nitrogen

AN content in intestinal digesta was determined by indigo phenol blue-spectrophotometry, according to Pu et al. [17]. The intestinal content was mixed with 0.2 mol/L hydrochloric acid at 1:9 (m:v) and stored at −20 °C. Before measuring, the mixture was thawed, then centrifuged at 10,000× *g* for 10 min at 4 °C, and the supernatant was taken for colorimetric analysis. The optimum conditions for the determination were 25 mg/L catalyst, water-soluble coloring at 40 °C for 20 min. The plate was read by a multi-functional enzyme labeling instrument (Spectra Max M5, Molecular Devices, San Jose, CA, USA) at 640 nm. There were 3 duplicates for each intestinal digesta sample, and 12 replicates in each group.

### 2.9. Fecal N and P

For N and P analysis, fecal samples were oven-dried at 65 °C for 72 h, then, those samples were crushed and sieved through a 100 mesh sieve. Fecal N was measured by transferring 0.2 g of each fecal sample into a digestive tube. Catalyst and concentrated sulfuric acid were added and digestion proceeded at 420 °C for 2 h, and then N was determined using an automatic nitrogen analyzer (8400, FOSS, Hillerod, Denmark). Fecal P was determined with the phosphorus vanadium molybdate yellow colorimetric method. First, 1.0 g sample was carbonized until smokeless, before being burned in a muffle oven at 550 °C for 6 h. After cooling, 10 mL 6 mol/L hydrochloric acid and a few drops of nitric acid were added, and the solution was boiled for 10 min, then made up to a volume of 100 mL with pure water. Finally, 1 mL of the solution was taken out for colorimetric analysis. The plate was read by a multi-functional enzyme labeling instrument (Spectra Max M5, Molecular Devices, San Jose, CA, USA) at 420 nm. There were 3 duplicates for each fecal sample, and 6 replicates in each group.

### 2.10. Statistical Analyses

Statistical analysis was computed using the Statistical Package for Social Sciences (SPSS) software, version 19.0. The results were analyzed using Duncan’s multiple range tests and one-way analysis of variance (ANOVA). Results are expressed as mean values and SEM, *p* ≤ 0.05 was considered significant, and 0.05 < *p* ≤ 0.10 indicated a trend.

## 3. Results

### 3.1. Animal Performance

Data on animal performance are reported in Table 2. Dietary treatments had no effect on initial BW, final BW, ADG, ADFI, or F/G (*p* > 0.05).

### 3.2. Plasma Biochemical Indicators

As shown in Table 3, compared with the CON group, the albumin content of the CE group was significantly increased by 18.8% (*p* = 0.011). The ratio of albumin to TP increased significantly (*p* = 0.015) in the CE and ANTI groups. There were no significant differences in plasma urea, NH_3_, PUN, or TP concentration among the three treatments (*p* > 0.05).

### 3.3. PFAA

The PFAA were determined and are described in Table 4. Compared with CON and ANTI groups, the CE group had higher concentrations of plasma total essential amino acids (EAA) (*p* = 0.003), threonine (*p* < 0.001), leucine (*p* = 0.054), histidine (*p* = 0.066), arginine (*p* = 0.094), valine (*p* = 0.094), and citrulline (*p* = 0.081). Compared with the CON group, CE increased α-amino adipic acid (*p* = 0.035) content, while compared with the ANTI group, CE increased 3-methylhistidine (*p* = 0.020) content.

### 3.4. Intestinal Morphology

The effect of CE on piglets’ intestinal morphology was assessed and is presented in Table 5. Compared with the CON group, the CD of duodenum (*p* = 0.004), jejunum (*p* = 0.013) and ileum (*p* < 0.001) decreased significantly, and the VCR of the duodenum (*p* = 0.057) and ileum (*p* < 0.001) increased in ANTI and CE groups. There were no significant differences in VH on the duodenum, jejunum or ileum among the three groups (*p* > 0.05).

### 3.5. Digestive Enzymes Activity

As shown in Table 6, compared with the CON group, the activity of AKP in duodenum was significantly increased in the CE and ANTI groups (*p* = 0.004). Duodenal lipase activity in the CE group was significantly higher than that in the CON and ANTI groups (*p* = 0.021). ANTI and CE tended to increase the activity of duodenal trypsin (*p* = 0.051). CE significantly increased jejunum AKP activity compared with the CON group (*p* = 0.041) and tended to increase the activity of lipase (*p* = 0.052). ANTI and CE groups significantly increased jejunum trypsin activity (*p* = 0.007).

### 3.6. AN in Intestinal Digesta

Intestinal digesta AN and fecal N and P emissions were determined and are shown in Table 7. Intestinal digesta AN and fecal N and P emissions of piglets fed with CE and ANTI were not significantly different (*p* > 0.05) compared with the CON.

## 4. Discussion

There are many studies that have suggest that plant extracts have a positive effect on the growth performance of piglets, such as extract from Nigella sativa L. and fenugreek seed [18,19]. Naringin, for one, improves the final BW and feed conversion rate of piglets [20]. However, one study suggested that no positive effect of hesperidin on the growth performance of broilers was observed [21]. Although our results show that ANTI and CE had no statistically significant effect on animal performance, the effects of ANTI and CE were better than those of the CON group, numerically.

TP and PUN are indicators for the overall metabolism of body proteins. Within a certain range, the higher the TP concentration, the stronger the body’s ability to synthesize and utilize proteins [22]. PUN concentration, on the other hand, has been negatively correlated with N deposition rate and protein utilization and a reduction in PUN indicates an increase in N utilization efficiency or a decrease in protein decomposition [23]. In this experiment, the PUN and TP concentrations in the plasma of piglets in the CE and ANTI groups were not significantly different to the CON group. PUN concentration has a close relationship with dietary crude protein concentration, but with a large enough increase of dietary crude protein intake, PUN concentration will plateau [24]. Therefore, with the crude protein of the basic diet identical among groups, the PUN concentrations did not appear to be affected by CE and ANTI and were within the normal range. Studies have suggested that plant extracts can promote digestion, absorption, growth and nutrient metabolism in piglets [25]. In this study, the levels of albumin, and albumin/TP in the CE group were significantly higher than those in the CON and ANTI groups, but urea nitrogen levels were not different among the groups. This implies that CE can promote protein deposition in piglets when protein metabolises while maintaining decomposition levels across treatments. Urea is the main N-containing bi-product of amino acid catabolism and has a strong linear relationship to the PUN concentration [26]. Compared with CON, piglets fed with CE had increases in urea circulating AA in the plasma and also had the highest urea content. The concentration of citrulline in the CE group increased by 30.3%–37.7% and the concentration of arginine increased by 18.9%–33.0%. Both arginine and citrulline participate in the urea cycle [27], so these results imply that CE may affect the urea cycle. In support of this, it has been shown that citrus naringin can regulate proteins involved in the urea cycle pathway [28]. While this means that CE might be able to affect kidney function or citrulline uptake, there was no direct effect on urea in our study. Under normal physiological conditions, blood NH_3_ concentration is maintained at a low level, but damage to the liver or kidney can lead to elevated blood NH_3_ [29]. Our results show that blood NH_3_ levels were not different among the three groups, suggesting that CE had no negative effects on the health of the piglets, which is consistent with Ramakrishnan and Vijayakumar [28], who found that there were no significant differences between rats given naringin and control rats.

PFAA levels can reflect not only nutritional status, but also inflammatory status and disease activity. Physiologically, AA absorption and metabolism by tissues depend on the concentration of PFAA [30]. From the perspective of nutritional metabolism, AA are directly and indirectly related to the entire metabolic pathway, and the distribution of PFAA reflects the total metabolic flow of nutrients and their metabolites to and from all tissues and organs [31]. PFAA are a primarily source of AA used for muscle protein biosynthesis, and a key indicator of protein turnover in the body [15]. In this study, the concentrations of plasma leucine, histidine, valine, arginine, threonine, and total EAA increased when CE was administered, which indicates that their utilization in protein synthesis and/or catabolism was increased. In other words, our results suggest that CE increased the metabolic requirements for EAA. The increase in the concentrations of EAA was likely due to an increase in EAA catabolism. An alternative explanation could be that the increase in EAA concentration resulted from increased bioavailability of EAA from the diet, perhaps because CE improved the digestion of N, and increased the availability of AA. To help approximate the fate of the excess AA, plasma 3-methylhistidine has been identified as a potential biomarker of muscle protein turnover [32]. Indeed, plasma 3-methylhistidine concentration was increased in the CE group, indicating that CE increased muscle protein synthesis or reduced protein mobilization. This is consistent with the increase of EAA in plasma of CE group. The mechanism by which CE acts is not yet clear, and further study is needed. In summary, the fact that increases in AA concentrations were mainly observed in CE group may indicates that CE is more beneficial to piglets than antibiotics.

VH, CD, and VCR are key indicators of the status of the intestinal barrier and are closely related to nutrient digestion and absorption. Increased villus diameter and VCR are indicative of a higher number of more functionally mature intestinal epithelial cells [33]. Increasing the VH and VCR can improve the intestinal absorption capacity, while a decrease of CD reflects an increase in the proliferation of intestinal epithelial cells [34]. Many studies have suggested that plant extract can promote intestinal development and improve intestinal morphology in animals. For example, feeding grape seed grape residue extract significantly increased the duodenal VCR in piglets [35]. In another example, compared with lipopolysaccharide-stimulated mice, Acanthopanax senticosus extract increased the VH and VCR of jejunum and ileum, and decreased jejunum CD [34]. Likewise, Lagenaria siceraria extract increased the height, width and area of jejunal villi in irradiated mice, and improved villi morphology and tight junction integrity [36]. In the present study, the VH (duodenum) and VCR (duodenum, ileum) of the CE and ANTI groups were higher than that of CON, while the CD (duodenum, jejunum, ileum) was lower, which is consistent with previous studies [34,35,36]. These measurements demonstrate the anti-inflammatory and anti-oxidant effects of CE flavonoids on intestinal repair and protection, and the overall improvement of intestinal morphology. Flavonoids are involved in the maintenance of the integrity of the intestinal tight junction barrier, which helps ensure the health of piglets [37]. Naringin supplementation, for example, was able to inhibit colonic inflammation and injury induced by sodium dextran sulfate, protecting the intestinal barrier in mice [38]. Likewise, citrus peel flavonoids increased the expression of the proteins Claudin-4 and Occludin in the ethanol-induced Caco-2 monolayer [37].

Maintaining normal intestinal digestion and absorption functions is important in preventing weaning stress. The digestion of proteins, fats, and carbohydrates can be achieved by proteases, lipases, and amylases. For example, enzymes such as sucrase, glucanase, lactase, and amylase and maltase in the intestinal mucosa are involved in sugar catabolism and affect the digestion and absorption of carbohydrates [39]. In this experiment, there was no change in the activities of sucrose, maltase, and amylase in duodenum and jejunum when CE and ANTI were administered. This suggests that CE and ANTI do not affect the digestion and absorption of carbohydrates. In addition, these results show that dietary supplementation with CE increased the activities of AKP, lipase, and trypsin in the duodenum and jejunum of piglets. Intestinal AKP is considered to be a key marker enzyme and plays an important role in the intestinal absorption of lipids [40], and lipase is responsible for the digestion of lipids [41]. These results again support the hypothesis that CE improves the digestion and absorption of fat and protein in piglets. At the same time, the observed improvement of intestinal digestion by CE supplementation was consistent with the improvements observed in the morphological comparison.

N emissions from pig excreta are NH_3_, ammonium ions and urea (with traces of nitrate and nitrite) [42]. NH_3_ and amines are thought to be harmful to intestinal health. These compounds, when in high concentrations in the intestine, can negatively affect the development of intestinal mucosa [43], which may be the cause of diarrhea in weaned piglets. CE reduced the AN concentration in the ileum and cecum digesta (7.8%–35.0%). This is may be caused by the extracts’ flavonoid compounds, an increase in N digestibility (enhancement of trypsin activity), or changes in microbial flora. Although the data are not statistically significant, it can still be used to guide animal production, and is worth investigating further.

In pig production, inefficient feed digestion can result in increased nutrient emissions into the environment. The nutrients of greatest environmental concern are N and P, which are usually supplied in excess in the diet [44]. Our study found that CE did not to reduce N and P emissions from feces, which is consistent with Panetta et al. [45], who observed no significant difference in AN content and N emission rate in feces when fed with Yucca extract. Similarly, Yucca extract did not reduce the concentration of total N, AN, P, or total ammonia in horse manure [46]. Reducing nitrogen and phosphorus emissions from feces is not a simple matter and more research is needed.

## 5. Conclusions

In brief, CE increased the absorption and utilization of N nutrients, improved intestinal morphology, and improved digestive enzymes activity, but had no effect on fecal N and P emissions. CE and antibiotics have similar effects in these aspects.

## Figures and Tables

**Table 1:** Composition and nutrient levels of basal diets (air-dry basis).

Ingredients (%)	Phase		Nutrient Levels (%) ^b^	Phase	
	d 1–14	d 15–28		d 1–14	d 15–28
Corn	35.31	47.55	Digestible energy (MJ/kg)	14.85	14.71
Extruded maize	15.00	13.00	Crude protein	19.20	19.10
Fermented soybean meal	9.00	8.50	Calcium	0.68	0.70
Peeled soybean meal	0.00	9.00	Total phosphorus	0.56	0.53
Expanded soybean	10.00	6.00	Available phosphorus	0.39	0.34
Fish meal	4.00	4.00	Lysine	1.57	1.41
Whey	11.00	6.00	Methionine + Cystine	0.89	0.81
Soybean hulls	5.00	0.00	Threonine	0.97	0.88
Soybean oil	1.20	0.00	Tryptophan	0.26	0.23
Plasma protein powder	3.00	0.00			
White granulated sugar	2.00	2.00			
Choline chloride	0.20	0.18			
Salt	0.45	0.45			
Calcium hydrogen Phosphate	0.62	0.60			
Stone powder	0.65	0.74			
l-lysine hydrochloride	0.60	0.54			
dl-methionine	0.22	0.20			
l-threonine	0.21	0.21			
l-tryptophan	0.04	0.03			
Premix ^a^	1.50	1.00			

^a^ 1 to 14 d premix for each kg of diet: VA 12,400 IU, VD_3_ 2800 IU, VE 30 IU, VK 5 mg, VB_12_ 40 μg, VB_1_ 3 mg, VB_2_ 10 mg, nicotinic acid 40 mg, D-pantothenic acid 15 mg, folic acid 1 mg, VB_6_ 8 mg, biotin 0.08 mg, FeSO_4_·H_2_O 120 mg, CuSO_4_·5H_2_O 16 mg, MnSO_4_·H_2_O 70 mg, ZnSO_4_·H_2_O 120 mg, CaI_2_O_6_ 0.7 mg, Na_2_SeO_3_ 0.48 mg. 15 to 28 d premix for each kg of diet: VA 12,400 IU, VD_3_ 2800 IU, VE 30 IU, VK 5 mg, VB_12_ 40 μg, VB_1_ 3 mg, VB_2_, 10 mg, nicotinic acid 40 mg, D-pantothenic acid 15 mg, folic acid 1 mg, VB_6_ 8 mg, biotin 0.08 mg, FeSO_4_·H_2_O 90 mg, CuSO_4_·5H_2_O 12 mg, MnSO_4_·H_2_O 53 mg, ZnSO_4_·H_2_O 90 mg, CaI_2_O_6_ 0.53 mg, Na_2_SeO_3_ 0.36 mg. ^b^ Calculated value. The values are expressed as percentage (%), except for digestible energy.

**Table 2:** Effects of citrus extract diets, on initial body weight, final body weight, average daily gain (ADG), average daily feed intake (ADFI) and feed to gain ratio (F/G) of piglets.

Items	CON ^1^	ANTI ^2^	CE ^3^	SEM ^4^	*p*-Value
Initial BW (kg)	8.4	8.5	8.3	0.08	0.301
Final BW (kg)	16.4	17.4	17.0	0.34	0.144
ADG (g)	284.9	316.6	313.0	14.83	0.332
ADFI (g)	597	620	605	17.16	0.652
F/G	2.1	2.0	1.9	0.09	0.407

^1^ CON: basal diet; ^2^ ANTI: CON + 75 g/t chlortetracycline; ^3^ CE: CON + 300 mL/t citrus extract; ^4^ SEM means standard error of the mean.

**Table 3:** Effects of citrus extract diets, on plasma urea, NH_3_, plasma urea nitrogen (PUN), albumin, total protein (TP) and albumin/TP of piglets.

Items	CON ^1^	ANTI ^2^	CE ^3^	SEM ^4^	*p*-Value
Urea (ng/μL)	137.2	136.9	158.5	10.89	0.300
NH_3_ (ng/μL)	2.0	2.7	1.7	0.58	0.571
PUN (mmol/L)	4.9	5.0	5.5	1.01	0.274
Albumin (g/L)	28.7 ^b^	31.3 ^a,b^	34.1 ^a^	3.87	0.011
TP (g/L)	53.8	51.6	53.9	2.89	0.113
Albumin / TP	0.5 ^b^	0.6 ^a^	0.6 ^a^	0.08	0.015

^a,b^ Means in the same row with different superscripts differ (*p* < 0.05). ^1^ CON: basal diet; ^2^ ANTI: CON + 75 g/t chlortetracycline; ^3^ CE: CON + 300 mL/t citrus extract; ^4^ SEM means standard error of the mean.

**Table 4:** Effects of citrus extract diets on plasma free amino acids (PFAA) of piglets (ng/μL).

Items	CON ^1^	ANTI ^2^	CE ^3^	SEM ^4^	*p*-Value
EAA ^5^					
Isoleucine	7.7	7.8	9.0	0.58	0.228
Leucine	13.6	13.2	15.7	0.73	0.054
Methionine	5.3	4.9	4.8	0.36	0.619
Lysine	17.6	16.8	20.4	1.69	0.322
Histidine	5.1	5.2	6.7	0.49	0.066
Arginine	11.8	10.5	14.0	1.56	0.094
Valine	13.2	11.2	15.0	1.11	0.094
Phenylalanine	9.8	9.5	11.1	0.62	0.190
Threonine	12.8 ^b^	15.2 ^b^	24.2 ^a^	5.54	<0.001
NEAA ^6^					
Aspartate	2.2	1.7	2.4	0.21	0.113
Serine	12.9	13.5	11.7	0.57	0.109
Glutamate	26.2	26.8	27.5	1.97	0.898
Glycine	59.0	68.7	57.5	4.77	0.235
Alanine	37.8	34.6	35.8	6.50	0.941
Cystine	5.2	5.3	6.3	0.56	0.337
Tyrosine	7.4	7.5	8.6	0.50	0.201
Proline	19.8	19.6	19.6	1.38	0.992
Hydroxy proline	15.1	15.6	12.5	1.04	0.110
β-alanine	2.8	2.9	2.6	0.25	0.740
Ornithine	6.1	6.5	7.3	0.47	0.207
Citrulline	6.3	6.0	8.3	0.68	0.081
Cystathionine	4.3	4.2	4.1	0.35	0.940
Phosphoserine	1.3	1.3	1.3	0.26	0.994
Taurine	17.6	19.1	21.0	0.51	0.342
α-amino adipic acid	6.8 ^b^	7.2 ^a,b^	8.9 ^a^	0.55	0.035
α-amino-n-butyric acid	1.5	1.9	2.1	0.19	0.101
1-methylhistidine	2.1	2.6	2.2	0.40	0.682
3-methylhistidine	1.8 ^a,b^	1.2 ^b^	2.9 ^a^	0.29	0.020
ΣEAA ^7^	96.3 ^b^	94.2 ^b^	120.6 ^a^	5.34	0.003
ΣNEAA ^8^	231.7	240.8	235.0	13.08	0.888
ΣAA ^9^	328.1	335.0	355.7	15.83	0.459

^a,b^ Means in the same row with different superscripts differ (*p* < 0.05). ^1^ CON: basal diet; ^2^ ANTI: CON + 75 g/t chlortetracycline; ^3^ CE: CON + 300 mL/t citrus extract; ^4^ SEM means standard error of the mean; ^5^ EAA: essential amino acids; ^6^ NEAA: non-essential amino acids; ^7^ ΣEAA = Total essential amino acids; ^8^ ΣNEAA = Total non-essential amino acids; ^9^ ΣAA = Total amino acids.

**Table 5:** Effects of citrus extract diets, on villus height (VH) and crypt depth (CD) of duodenum, jejunum, and ileum in piglets.

Items	CON ^1^	ANTI ^2^	CE ^3^	SEM ^4^	*p*-Value
Villus height (μm)					
Duodenum	370.3	334.5	415.4	56.76	0.093
Jejunum	377.0	396.4	369.3	52.10	0.585
Ileum	308.0	370.6	347.8	65.87	0.189
Crypt depth (μm)					
Duodenum	447.4 ^a^	375.1 ^b^	391.9 ^a,b^	40.62	0.004
Jejunum	293.4 ^a^	243.7 ^b^	232.5 ^b^	37.05	0.013
Ileum	267.8 ^a^	189.8 ^b^	191.1 ^b^	24.71	<0.001
Villus height/crypt depth					
Duodenum	0.9	1.0	1.1	0.15	0.057
Jejunum	1.5	1.7	1.7	0.33	0.389
Ileum	1.2 ^b^	2.1 ^a^	2.0 ^a^	0.37	<0.001

^a,b^ Means in the same row with different superscripts differ (*p* < 0.05). ^1^ CON: basal diet; ^2^ ANTI: CON + 75 g/t chlortetracycline; ^3^ CE: CON + 300 mL/t citrus extract; ^4^ SEM means standard error of the mean.

**Table 6:** Effects of citrus extract diets, on alkaline phosphatase (AKP), lipase, α-amylase, sucrose, maltase, pepsin and trypsin of duodenum and jejunum in piglets (U/mgprot).

Items	CON ^1^	ANTI ^2^	CE ^3^	SEM ^4^	*p*-Value
Duodenum					
AKP	2119.7 ^b^	2668.8 ^a^	2983.2 ^a^	141.33	0.004
Lipase (U/gprot)	162.5 ^b^	170.9 ^b^	198.1 ^a^	7.96	0.021
α-amylase	0.3	0.3	0.3	0.01	0.202
Sucrase	72.6	75.3	79.1	6.48	0.815
Maltase	193.7	156.8	197.1	41.78	0.774
Pepsin	0.9	1.1	1.0	0.15	0.730
Trypsin	25.0	43.9	40.0	4.96	0.051
Jejunum					
AKP	1973.2 ^b^	2207.2 ^a,b^	2703.7 ^a^	173.41	0.041
Lipase (U/gprot)	146.8	159.0	178.9	7.73	0.052
α-amylase	0.2	0.3	0.3	0.01	0.188
Sucrase	61.1	66.3	69.8	6.70	0.677
Maltase	59.6	47.0	60.7	15.60	0.806
Pepsin	0.4	0.8	0.4	0.16	0.250
Trypsin	7.1 ^b^	43.8 ^a^	41.0 ^a^	7.13	0.007

^a,b^ Means in the same row with different superscripts differ (*p* < 0.05). ^1^ CON: basal diet; ^2^ ANTI: CON + 75 g/t chlortetracycline; ^3^ CE: CON + 300 mL/t citrus extract; ^4^ SEM means standard error of the mean.

**Table 7:** Effects of citrus extract diets, on intestinal digesta ammonia nitrogen (AN), fecal nitrogen (N) and phosphorus (P) emissions of piglets.

Items	CON ^1^	ANTI ^2^	CE ^3^	SEM ^4^	*p*-Value
Ammonia nitrogen (mg/100 g)					
Ileum	55.9	44.0	36.3	13.36	0.626
Cecum	198.7	209.5	183.2	23.30	0.725
Fecal N and P emissions (%)					
N	4.4	4.4	4.5	0.14	0.979
P	0.9	0.9	1.0	0.09	0.840

^1^ CON: basal diet; ^2^ ANTI: CON + 75 g/t chlortetracycline; ^3^ CE: CON + 300 mL/t citrus extract; ^4^ SEM means standard error of the mean.
